# The Geography of Diabetes among the General Adults Aged 35 Years and Older in Bangladesh: Recent Evidence from a Cross-Sectional Survey

**DOI:** 10.1371/journal.pone.0110756

**Published:** 2014-10-30

**Authors:** Md. Mobarak Hossain Khan, Oliver Gruebner, Alexander Kraemer

**Affiliations:** 1 Department of Public Health Medicine, School of Public Health, Bielefeld University, Bielefeld, Germany; 2 Department of Epidemiology, Mailman School of Public Health, Columbia University, New York, New York, United States of America; The Ohio State University, United States of America

## Abstract

**Objective:**

To report geographical variations of sex-specific diabetes by place of residence (large cities/city corporations, small towns/other urban areas, rural areas) and region of residence (divided into seven divisions) among general adults (35+ years of age) in Bangladesh.

**Methods:**

The recent cross-sectional data, extracted from the nationally representative Bangladesh Demographic and Health Survey 2011, was used. A total of 3,720 men and 3,823 women aged 35+ years, who participated in the fasting blood sugar testing, were analysed. Any person with either fasting plasma glucose level (mmol/L) ≥7.0 or taking medication for diabetes was considered as a person with diabetes.

**Results:**

The prevalence of diabetes was 10.6% in men and 11.3% in women. Bivariable analyses indicated significant variations of diabetes by both geographical variables. The prevalence was highest in city corporations (men 18.0%, women 22.3%), followed by small towns (men 13.6%, women 15.2%) and rural areas (men 9.3%, women 9.5%). Regional disparities in diabetes prevalence were also remarkable, with the highest prevalence in Chittagong division and lowest prevalence in Khulna division. Multivariable logistic regression analyses provided mixed patterns of geographical disparities (depending on the adjusted variables). Some other independent risk factors for diabetes were advancing age, higher level of education and wealth, having TV (a proxy indicator of physical activity), overweight/obesity and hypertension.

**Conclusions:**

Over 10% of the general adults aged 35 years and older were having diabetes. Most of the persons with diabetes were unaware of this before testing fasting plasma glucose level. Although significant disparities in diabetes prevalence by geographical variables were observed, such disparities are very much influenced by the adjusted variables. Finally, we underscore the necessities of area-specific strategies including early diagnosis and health education programmes for changing lifestyles to reduce the risk of diabetes in Bangladesh.

## Introduction

Although diabetes was mainly a disease of Western and affluent societies [1], it is now a global public health problem. Globally, the number of persons (aged 20–79 years) with diabetes is projected to increase from 285 million in 2000 to 439 million in 2020, mostly in low- and middle-income countries [2]. The global rank of diabetes as a leading cause of disease burden moved from 21 in 1990 to 14 in 2010 [3]. Similarly, the global position of diabetes as a mortality risk factor shifted from 15 in 1990 to 9 in 2010 [4]. Asia is emerging as the epicentre of diabetes, which contains over 60% of the global cases with diabetes [1]. In this region, the number of persons with diabetes within the age group of 20–79 years is projected to increase from approximately 114 million in 2007 to 180 million in 2025 [5]. Substantial demographic, socioeconomic and epidemiologic changes in recent decades particularly in some of the most populous countries in Asia [6] – indicated by rapid urbanisation, economic growth, modernisation, population aging, sedentary lifestyles, rising obesity and consumption of fast and fatty foods – have increased the total burden of diabetes in this region [2], [7]–[12].

Like other low- and middle-income countries in South Asia, Bangladesh – the eighth most populous country (with more than 153 million population) in the world [13]– is also observing the rising prevalence of diabetes [12], [14]. The pooled prevalence of type 2 diabetes increased from 3.8% in 1995–2000 to 5.3% in 2001–2005 and to 9.0% in 2006–2010 [14]. This country is predicted to be one of the top 10 countries worldwide with 10.4 million cases with diabetes in 2030 in the age group of 20–79 years, which were approximately 5.7 million in 2010 [2]. Such a rapid increase in diabetes can be attributed to remarkable socio-economic, demographic and epidemiologic changes, which Bangladesh has undergone over the last few decades [13], [15], [16]. Particularly the burden of disease has shifted remarkably from communicable to non-communicable diseases [13], [17]. Increasing life expectancy, rapid urbanisation and urban lifestyles, declining poverty, rising literacy and increasing female employment opportunities mainly in the garment industries also influence the pace of above-mentioned changes [15], [16], [18]–[20].

Urban-rural differences in diabetes are reported in many countries including Bangladesh [8], [12], [21]–[25]. Generally urban populations reveal more risk factors of diabetes than rural population because of the differences in lifestyles and nutritional pattern. For instance, the prevalence of hypertension is higher in urban than rural areas [24]. The urban population is more obese than the rural population [18], [25]. Urban lifestyles can lead to changes in dietary habits and physical activities [10]. Urbanisation is found to be positively associated with the intake of increased fat, oil and animal-based foodstuffs, which can increase the bodyweight [1], [18], [26]. Migration from rural to urban areas is related with obesity, glucose intolerance and dyslipidaemia [8], [26]. Generally, rural populations are engaged in physically vigorous manual/agricultural/traditional activities, which protect them from developing glucose intolerance. In contrast, urban populations are mostly engaged in light and sedentary occupations requiring less physical activity. Consequently, urban populations practicing sedentary lifestyles and adapting western lifestyles have higher calories and fat intake than rural populations [1], [21], [27], [28].

The prevalence of diabetes also varies by socio-economic status, genetic and environmental factors [8]. Advancing age, wealthy class and well-educated people suffer more from diabetes than poor and illiterate people [8], [12], [22], [27]–[30]. Since higher income groups in low-income countries have a higher ability to purchase higher caloric foods, they are associated with higher intake of calories, fat and sugar, which can increase the prevalence of diabetes and other metabolic syndromes [8], [12], [31]. Obesity/overweight - which is rapidly increasing in Asia including Bangladesh - is strongly associated with diabetes due to insulin resistance [5], [10], [12], [14], [28], [30], [32]–[35]. Other factors also increase the risk of diabetes - low intake of fruits and vegetables [14], family history of diabetes, physical inactivity and sedentary lifestyles [8], [12], [14], [27], [30], [32], [36], increasing prevalence of smoking [1], [14], stress and depression [5], [14] and hypertension [12], [14], [21], [22], [30], [32], [33], [36], [37].

The public health consequences of diabetes are well-reported. It can increase the risk of cardiovascular and peripheral vascular disease including stroke. It can also cause various long-term complications like blindness, renal failure, amputations and various cancers [5], [38]. An increasing prevalence of diabetes in the population and its long-term financial, social and health consequences can pose a serious challenge for the health system in Bangladesh and other low- and middle-income countries [10], [25]. These challenges might be more in Bangladesh since this country is also grappling with malnutrition, infectious diseases and poor healthcare facilities [31]. It is also important to identify the risk factors for diabetes, which can be avoided/modified through proper knowledge and lifestyle changes [32], [37], [38]. In order to assist policymakers and health managers, geographically representative studies based on large samples are imperative [14]. Until recently research on general adults having diabetes (35+ years) using large-scale representative samples are still scarce in Bangladesh [33]. Although some studies investigated rural-urban disparities in diabetes (mostly based on small scale data), studies focusing on regional disparities in diabetes prevalence across the whole nation are scarce in Bangladesh. We aimed at (i) describing the prevalence of diabetes in Bangladesh, (ii) identifying geographical disparities in diabetes, and (iii) investigating exposure factors for diabetes in the general adult population (35+ years of age) of Bangladesh.

Specifically, we hypothesized (i) diabetes varies significantly between large cities/city corporations (highest risk) to small towns/other urban areas to rural areas (lowest risk), and (ii) that the prevalence/risk of diabetes differs from one region to another due to socio-demographic and socio-economic disparities.

## Methodology

### Data source: Bangladesh Demographic and Health Survey (BDHS) 2011

The most recent data sets of the Bangladesh Demographic and Health Survey (BDHS) 2011 were used for this study. The BDHS 2011 was the sixth round survey with previous surveys of 1993–94, 1996–97, 1999–2000, 2004, and 2007. It was conducted under the authority of the National Institute of Population Research and Training (NIPORT) of the Ministry of Health and Family Welfare. The ICF International, located in Calverton, Maryland, provided all technical assistance to the survey [39].

### Types of questionnaires employed in BDHS 2011

Five types of questionnaires namely for households, women, men, communities, and causes of death among under-five children (verbal autopsy) were used. The MEASURE DHS model questionnaires were the basis of the household and individual questionnaires, which were first drafted by the technical working group of local and international experts to use in Bangladesh. Then the draft questionnaires were circulated to other interested groups for further feed-back. Finally, the experienced technical review committee reviewed all feedbacks and finalised the English questionnaires, which were translated into Bangla for data collection [39].

### Data extraction

MEASURE DHS produced separate data files for BDHS 2011 for distribution using flat or hierarchical formats. Some of these files are named as household, household member, women (ever married aged 12–49 years) and men (ever married aged 15–54 years) data files. We converted these files into SPSS data files for analyses. In this study, we used the household member data set, which has the highest sample size. Other two relevant data sets namely men and women data were restricted to the age cohort (35–49 for women and 35–54 for men) and marital status (married women). As a result, the sample was reduced by almost 50%. The data file contains some basic information for each household member including age, sex, education and relationship to the head of the household. Information on floor and wall materials of the house, sources of water, type of toilet facilities, and ownership of consumer goods were also collected. The main purpose of the household questionnaire was to identify eligible women and men for individual interviews as well as for anthropometric measurements for body mass index (BMI), anaemia, blood pressures (systolic and diastolic) for hypertension and fasting plasma glucose levels for diabetes [39].

**Figure 1:**
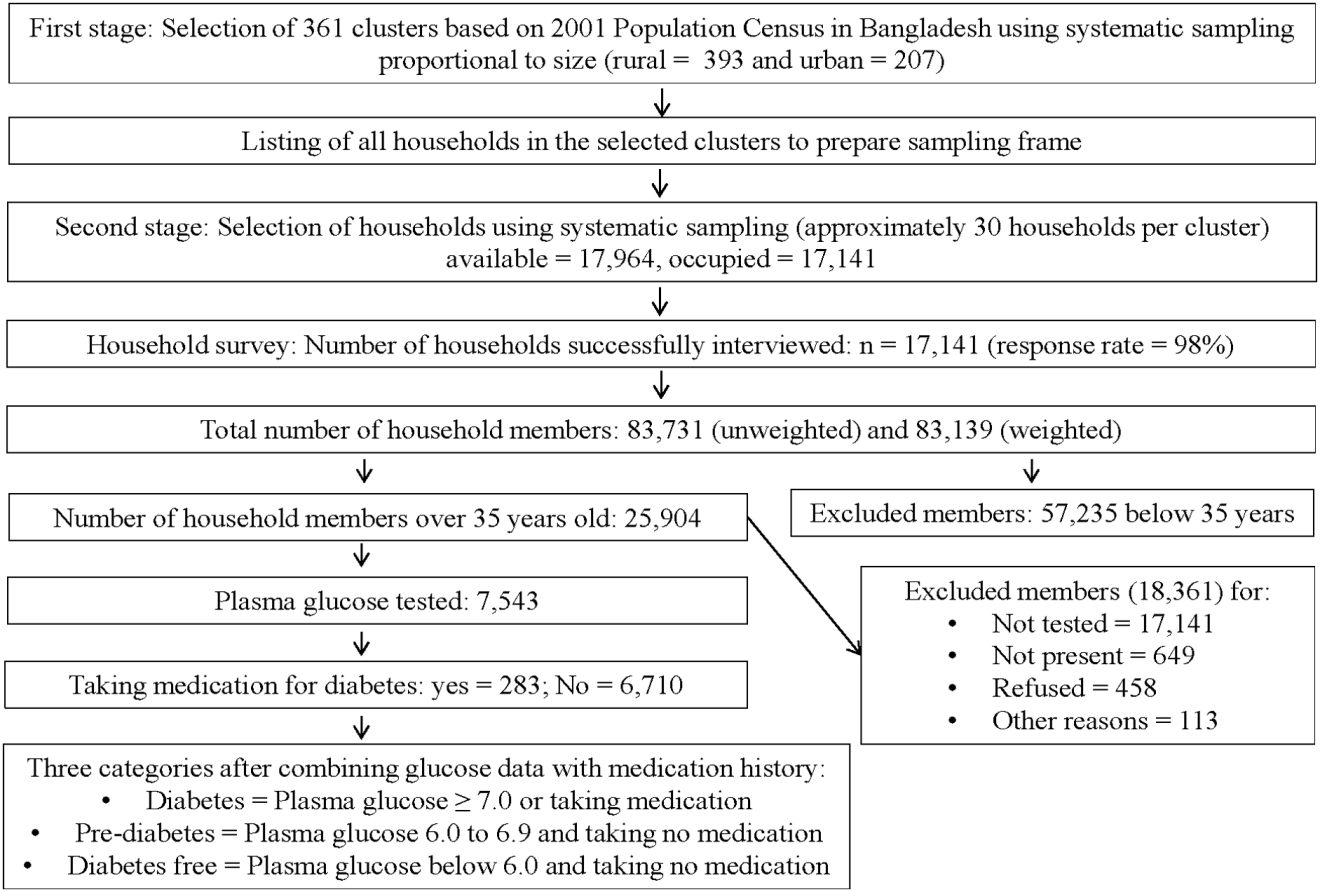
Sampling strategy and study population.

The unweighted number of household members was 83,731, which was reduced to 83,139 after weighting (based on household sample weight expressed with six decimal). Since fasting blood glucose was measured only for the 35 years and older subjects, we excluded all subjects below 35 years of age. As a result, our number of subjects was reduced to 25,904 aged 35 years and older. We again excluded those subjects who were not tested for fasting blood glucose (17,141), who were not present during testing (649), who refused to participate in testing (458) or who were missing for some other reasons (113). Such exclusions reduced our subjects to a number of 7,543, which was the final sample for this study. In addition to fasting plasma glucose testing, all these people were also asked to mention whether they were taking any medication for diabetes (Figure 1).

### Ethical issues and verbal consent

The 2011 Bangladesh Demographic and Health Survey (BDHS) was reviewed and approved by the ICF Macro Institutional Review Board (USA), which complies with all of the requirements of 45 CFR 46 “Protection of Human Subjects”. The 2011 Bangladesh DHS was also reviewed and approved by the National Research Ethics Committee of the Bangladesh Medical Research Council (Dhaka, Bangladesh).

From each participant of the survey, verbal consent was also taken before starting the interview/data collection. Since blood taking from people is like a human experiment, everyone selected for testing fasting blood sugar was asked for informed consent. The survey team visited all people twice. At the first visit/day, the survey team informed the participants that sugar blood testing is a part of the survey and a high level of blood sugar may increase the risk of heart disease and stroke. It was also explained that the team will only collect a few drops of blood from a finger by clean and completely safe equipment, which had never been used before and which will be thrown away after one time use. Furthermore, it was communicated that the collected blood will be tested for glucose immediately and the result with possible explanations (e.g. normal/high glucose level) and suggestions (e.g. to consult a health facility or doctor for high level) will be given to the participant right away. It was further mentioned that blood-testing results will be kept strictly confidential and will not be shared with anyone except member of the survey team. Having received a positive answer (i.e. verbal content) from the selected participants at the first visit, blood samples were collected at the second visit/day [39]. All these information were anonymized and de-identified prior to analysis.

### Variables

#### Dependent variable

The dependent variable was having diabetes. Having diabetes was defined as having a fasting plasma glucose level of ≥7.0 (mmol/L) or diabetes medication at the time of survey. Any person having either fasting plasma glucose (mmol/L) between 6.0 to 6.9 and no diabetes medication at the survey was defined as pre-diabetes person. Similarly, any person having either fasting plasma glucose (mmol/L) below 6.0 and no diabetes medication was defined as diabetes free person. For analytical purposes, we merged two groups (pre-diabetes and diabetes free) together to make the variable dichotomous (person with diabetes versus no diabetes).

#### Independent variables

The following variables were used as independent variables for detailed analyses (with categories in parenthesis):

Sex (male, female), place of residence (city corporation/large cities, small town/other urban area, rural), region of residence (Barisal, Chittagong, Dhaka, Rajshahi, Rangpur, Khulna, Sylhet), age (35–44, 45–54, 55–64, 65+), education (no, primary, secondary, higher secondary+), current marital status (currently married, widowed/divorced/separated/deserted/never married), having TV (no, yes), currently working (no, yes), having bicycle (no, yes), body mass index (underweight, normal, overweight), wealth index based on principal component analysis (poorest/poorer, middle, richer/richest) and combined status of hypertension (non-hypertensive/pre-hypertensive, hypertensive). It should be noted that having TV, currently working and having bicycle were used as proxies of physical mobility. To create the combined status of hypertension, we utilized the collected information of systolic blood pressure (SBP) and diastolic blood pressure (DBP). According to the report of Chobanian et al [40], hypertension categories are:

- Being hypertensive: SBP ≥140 mmHG (millimetres of mercury) or DBP ≥90 mmHG or taking hypertensive medication
- Being pre-hypertensive: SBP 120–139 mmHG or DBP 80–89 mmHG or not taking hypertensive medication
- Being non-hypertensive: SBP <120 mmHG or DBP <80 mmHG or not taking hypertensive medication.

For multivariable analyses, we combined being non-hypertensive and pre-hypertensive together. Our main target was to assess the geography of diabetes focusing on two spatial variables - place of residence (city corporation/large cities, small town/other urban area, rural) and region of residence (Barisal, Chittagong. Dhaka, Khulna, Rajshahi, Rangpur, Sylhet). Key analyses were stratified for sex. In addition to the above-mentioned variables, some descriptive information regarding diabetes is also provided (Table 1).

**Table 1:** Diabetes related information among adults in Bangladesh.

Questions/variables	Categories	Men		Women		Total	
		*N = 3720	Valid%	*N = 3823	Valid%	*N = 7543	Valid%
Q1: Have you ever heard an illness called diabetes?	No	150	4.1	377	9.9	527	7.0
	Yes	3557	95.6	3437	90.1	6994	92.7
Q2: Have you ever been told by a doctor or nurse that you have diabetes?[only valid for yes in Q1]	No	3369	94.8	3206	93.4	6575	94.1
	Yes	186	5.2	227	6.6	413	5.9
Q3: Are you taking medication for diabetes prescribed by a doctor or nurse?[only valid for yes in Q1]	No	3429	96.4	3280	95.5	6709	96.0
	Yes	127	3.6	156	4.5	283	4.0
Q4: How do you take the medication? [only valid for yes in Q1]	Injected	22	18.2	26	16.9	48	17.5
	Orally	95	76.7	112	73.1	207	74.7
	Both	6	5.2	15	10.0	22	7.9
V1: Diabetes status based on fasting plasma glucose level	Diabetes free	2391	64.3	2479	64.8	4870	64.6
	Pre-diabetes	974	26.2	975	25.5	1950	25.9
	Diabetes	354	9.5	368	9.6	723	9.6
V2: Diabetes status based on either fasting plasma glucose and history of taking medication	Diabetes free	2371	63.8	2445	64.0	4816	63.9
	Pre-diabetes	952	25.6	948	24.8	1900	25.2
	Diabetes	396	10.6	430	11.3	826	11.0
V3: Binary status of diabetes based on either plasma glucose and history of taking medication	No diabetes	3324	89.4	3393	88.7	6716	89.0
	Diabetes	396	10.6	430	11.3	826	11.0

*N is not always same for missing and system missing information.

### Statistical analysis

A series of analysis has been performed ranging from simple frequency analysis to multivariable logistic regression analysis. Descriptive information based for the study sample on the selected variables is provided first.

Then cross-table (i.e. bivariable) analysis was performed to test the association of selected categorical variables with the dependent variable. We also provided simple correlation coefficients between diabetes status (yes, no) for each of the selected independent variables. Any variable, which showed a significance level of P≤0.10 (for the correlation coefficient) was considered for the multivariable logistic regression model. Using multivariable logistic regression, we mainly calculated the odds ratio (OR) and 95% confidence interval (CI) for each independent variable. Our logistic regression models ranged from simple logistic regression to multivariable models. The first two models were simple logistic regressions (one for rural-urban place of residence and one for region of residence) which included only one independent variable. Thereafter, the independent variables were manually added to the model one by one, while checking for their statistical significance and their effect on the primary independent variable. Finally, we had 11 statistical models for male respondents (from IM to XIM where ‘M’ indicates models for men) and nine models for female respondents (from IW to IXW where ‘W’ indicates models for women). Since some independent variables could suffer from the problem of multicollinearity, we checked this problem (through variance inflation factor - VIF) before entering variables into the multivariable models. The VIFs ranged from 1.05 to 2.59 (male) and from 1.02 to 2.49 (female).

## Results

### Diabetes related information including prevalence

Table 1 reveals that more than 90% (both sexes) of the adults aged 35 years or more had ever heard of diabetes with slightly higher rate among men than women. Around 6% of the adults had ever been told by a doctor or nurse that they had diabetes (men 5.2% and women 6.6%). Only 4% of the informed adults (both sexes) having diabetes were taking medicine (mostly orally) for diabetes. The prevalence of diabetes (based on the fasting plasma glucose testing) was around 9.6% for the total sample. Similarly, the prevalence of pre-diabetes for the combined sample was 25% (men 25.6% and women 24.8%). Combining both fasting plasma glucose outcomes and being on diabetes treatment, the overall prevalence of diabetes was 11.0% with slightly higher prevalence among women (11.3%) as compared to men (10.6%) (p = 0.402). Out of 390 men and 405 women aged 35 years and older with diabetes, 67.4% and 61.5% were not on medication. Similarly, out of the total sample who were on medication, only 32.6% men and 39.7% women had fasting plasma glucose below 7.0 (not shown in table).

### Geographical disparities in diabetes prevalence

**Figure 2:**
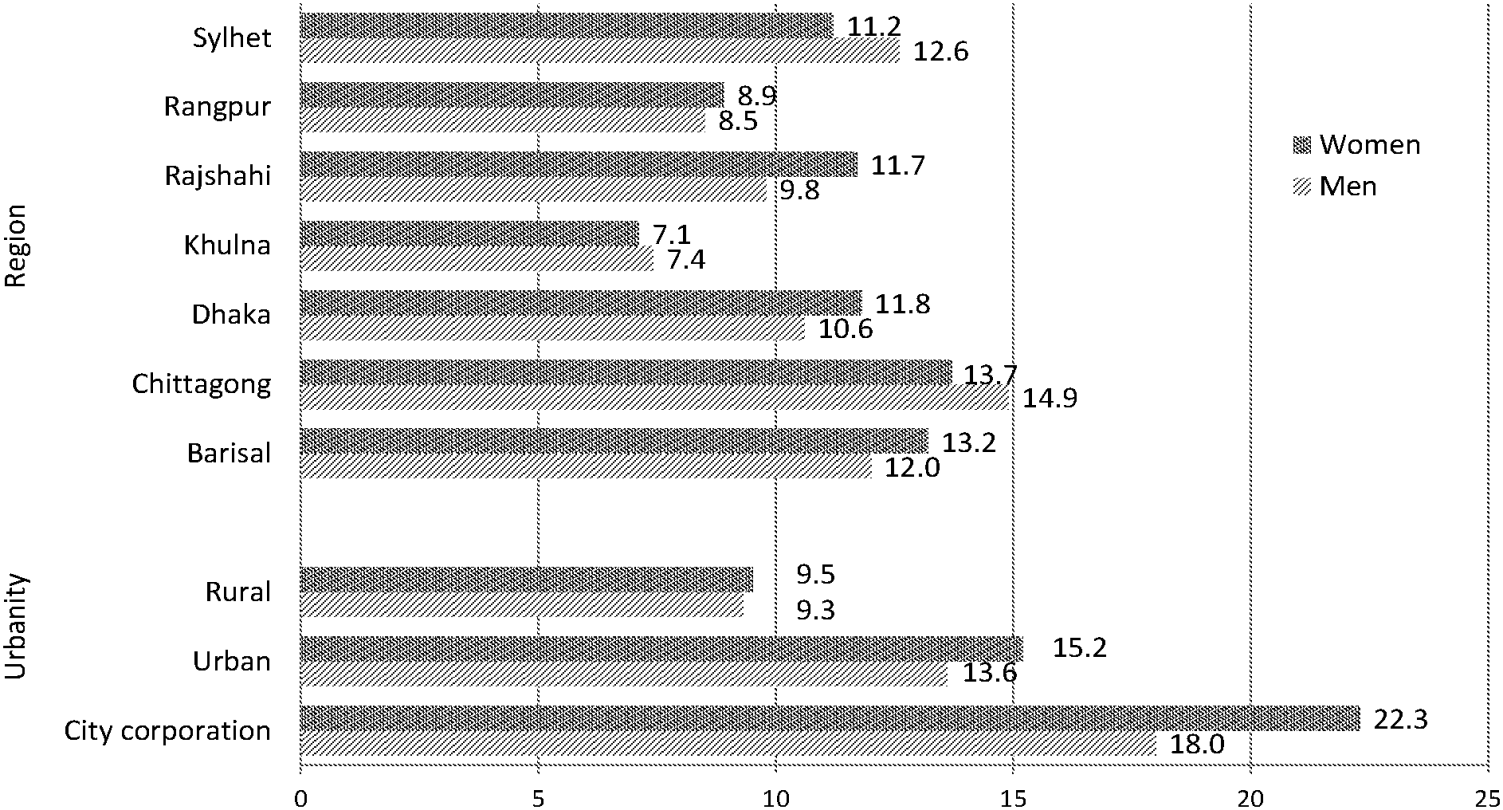
Prevalence of diabetes by urbanity and region of residence.

The sex-specific prevalence of diabetes was compared by place of residence (city corporations/large cities, other urban areas/small towns, rural areas) and region divided into seven administrative divisions (Figure 2). Although the regional variation of diabetes was comparable for both sexes, the variation of diabetes by place of residence was more prominent for women than men. The prevalence (overall and sex-specific) was significantly higher in city corporations (overall = 20.1%, men = 18.0%, women = 22.3%) and urban areas (overall = 14.4%, men = 13.6%, women = 15.2%) as compared to rural areas (overall = 9.4%, men = 9.3%, women = 9.5%) (P<0.001). Regional differences of diabetes were also remarkable, ranging from around 7.1% in Khulna division to 13.7% in Chittagong division for women (P<0.012) and from 7.4% in Khulna to 14.9% in Chittagong for men (P<0.002).

### Disparities in diabetes prevalence by adjusted variables

Sex-specific prevalence of diabetes by selected factors representing socio-demographic and hypertension statuses are presented in Table 2. Diabetes was more frequent in the age group of 55–64 years (men 16.2% and women 13.3%) as compared to other age groups. A rising trend was observed up to 64 years of age, which then declined sharply mainly for men. However, the disparities in diabetes prevalence were significant only for men (P<0.001). Education, wealth index and BMI showed strong positive associations with diabetes for both sexes. For example, subjects with 11+ years of education indicated higher prevalence of diabetes (men 21.5% and women 22.6%) as compared to no (men 7.8% and women 8.7%) and other educational groups. Similarly, participants with overweight/obesity had higher prevalence of diabetes (men 21.0% and women 21.6%) than participants with underweight (men 8.0% and women 5.9%) and normal weight (10% for both sexes) groups. Currently married men had significantly lower prevalence of diabetes as compared to other group composed of married but presently single and never married men (P = 0.019). Household having television was also positively associated with diabetes for both sexes. Having bicycle was a significant factor only for men (p = 0.011) but not for women (p = 0.202). Men who were working at the time of survey had a significantly (p<0.001) lower prevalence of diabetes (9.7%) as compared to non-working men (16.0%). Ownership of agricultural land was positively associated with women diabetes only (p = 0.009). Being hypertensive was also positively associated with diabetes for both sexes (p<0.001).

**Table 2:** Sex-specific prevalence of diabetes by selected factors indicating socio-demographic and hypertension status of the respondents.

Variables		Men			Women		
		N	Diabetes (%)	p+++	N	Diabetes (%)	p+++
	Total	3720	10.6		3823	11.3	
Age (in years)	35–44	1199	7.4	<0.001	1500	9.9	0.134
	45–54	1188	10.4		1025	11.4	
	55–64	616	16.2		671	13.3	
	65+	716	11.7		625	11.8	
Education (in years)	No	1402	7.8	<0.001	2237	8.7	<0.001
	1–5	1044	9.9		971	12.6	
	6–10	827	10.5		482	17.2	
	11+	446	21.5		133	22.6	
Marital status	Currently married	3608	10.4	0.011	2746	11.1	0.738
	Else+	111	18.0		1076	11.5	
Household having TV^1^	No	2143	8.0	<0.001	2193	7.9	<0.001
	Yes	1576	14.3		1631	15.7	
Currently working^1^	No	537	16.0	<0.001	3396	11.5	0.159
	Yes	3183	9.7		423	9.2	
Household having bicycle^1^	No	2591	11.5	0.011	2757	10.8	0.202
	Yes	1128	8.7		1065	12.3	
Own agricultural land	No	1780	10.4	0.633	1807	9.9	0.009
	Yes	1940	10.9		2015	12.5	
BMI	Underweight	627	8.0	<0.001	707	5.9	<0.001
	Normal	2656	10.0		2332	10.0	
	Overweight/obese	328	21.0		653	21.6	
Wealth index	Poorest/poorer	1461	7.7	<0.001	1450	6.9	<0.001
	Middle	723	7.3		770	7.9	
	Richer/richest	1536	15.0		1604	16.8	
Status of hypertension++	Non-hypertensive	1972	9.0	<0.001	1545	7.2	<0.001
	Pre-hypertensive	1012	10.4		1052	10.6	
	Hypertensive	725	15.7		1221	16.8	

+ Else: Divorced or widowed or deserted or separated or never married.

++ Based on three variables namely history of hypertensive medication, SBP and DBP.

+++ Based on Chi-square test.

1 Used as proxies of physical mobility.

### Correlation between diabetes and potential exposure factors

The correlation coefficients for diabetes and selected categorical variables are shown in Table 3. Diabetes was significantly positively correlated with age, education, having TV, wealth index, BMI and hypertension for both sexes. Some of the correlations of diabetes (namely with having TV, wealth index, BMI and hypertension) were stronger for women than men. The correlation coefficient between age and diabetes was comparatively stronger for men as compared to women. Diabetes was significantly negatively associated with the status of currently married (yes), currently working (yes) and having bicycle (yes) for men only. Ownership of agricultural land (yes) was only positively associated with diabetes for female.

**Table 3:** Bivariate correlation of diabetes with selected independent variables.

	Age	Education	Currently married	Having TV	Currently working.	Wealth index	Owner. agricul. land	BMI	Having bicycle	Status of hyper.
Male										
r	0.070	0.113	0.040	0.101	−0.081	0.122	0.008	0.090	−0.042	0.078
p-value	<0.001	<0.001	0.015	<0.001	<0.001	<0.001	0.616	<0.001	0.010	<0.001
Female										
r	0.031	0.116	0.006	0.121	−0.012	0.156	0.042	0.149	0.021	0.127
p-value	0.056	<0.001	0.710	<0.001	0.459	<0.001	0.009	<0.001	0.200	<0.001

### Multivariable logistic regression analysis

Summary results of multivariable analyses (based on various models) are shown in Table 4 (for men) and Table 5 (for women). Unadjusted results (models 1 M and IIM in table 4 and models 1F and IIF in table 5) showed significantly higher risks of diabetes in city corporations (OR_men_ = 2.13, 95% CI = 1.52–3.00 and OR_women_ = 2.72, 95% CI = 1.98–3.74) and small towns (OR_men_ = 1.54, 95% CI = 1.18–1.99 and OR_women_ = 1.71, 95% CI = 1.32–2.20) as compared to rural areas. Men from Chittagong and Barisal divisions showed higher risks of diabetes (Table 4) as compared to men from Dhaka division. Women from Khulna division always had significantly lower risks of diabetes. In contrast, women from Chittagong and Barisal divisions indicated higher risks of diabetes, these risks were insignificant when compared with Dhaka (Table 5).

**Table 4:** Results of simple and multivariable logistic regression analyses for male diabetes in Bangladesh.

		IM	IIM	IIIM	IVM	VM	VIM	VIIM	VIIIM	IXM	XM	XIM
Residence	City corporation vs rural (OR)	**2.13** ***	-	**2.21** ***	**1.58** *	**1.59** *	1.39	1.41	1.33	1.13	1.08	1.06
	95% CI	**1.52–3.00**		**1.55–3.15**	**1.09–2.30**	**1.10–2.32**	0.95–2.04	0.96–2.08	0.90–1.97	0.75–1.69	0.72–1.63	0.70–1.60
	Urban vs rural (OR)	**1.54** ***	-	**1.62** ***	**1.37** *	**1.37** *	1.22	1.23	1.19	1.07	1.04	1.04
	95% CI	**1.18–1.99**		**1.24–2.11**	**1.04–1.80**	**1.04–1.80**	0.92–1.63	0.92–1.63	0.89–1.59	0.79–1.45	0.76–1.41	0.76–1.41
Region	Barisal vs Dhaka (OR)	-	1.15	1.31	1.27	1.26	1.34	1.38	1.37	1.34	1.38	1.39
	95% CI		0.73–1.81	0.83–2.09	0.80–2.03	0.79–2.01	0.84–2.15	0.86–2.21	0.85–2.19	0.83–2.18	0.85–2.25	0.86–2.26
	Chittagong vs Dhaka (OR)	-	**1.46** **	**1.51** **	**1.59** **	**1.59** **	**1.61** **	**1.57** **	**1.57** **	**1.53** **	**1.53** **	**1.54** **
	95% CI		**1.09–1.95**	**1.12–2.03**	**1.18–2.16**	**1.17–2.15**	**1.19–2.18**	**1.16–2.14**	**1.16–2.13**	**1.11–2.09**	**1.12–2.10**	**1.13–2.11**
	Khulna vs Dhaka (OR)	-	**0.68** *	0.75	0.75	0.76	0.76	0.75	0.81	0.75	0.76	0.75
	95% CI		**0.46–0.99**	0.51–1.10	0.51–1.11	0.51–1.12	0.51–1.12	0.51–1.12	0.54–1.21	0.50–1.14	0.50–1.14	0.50–1.13
	Rajshahi vs Dhaka (OR)	-	0.91	1.10	1.12	1.12	1.15	1.14	1.18	1.20	1.22	1.22
	95% CI		0.65–1.27	0.78–1.55	0.79–1.58	0.79–1.59	0.81–1.63	0.80–1.62	0.83–1.69	0.83–1.73	0.85–1.75	0.85–1.75
	Rangpur vs Dhaka (OR)	-	0.78	0.91	0.98	0.97	1.01	0.98	1.06	1.02	1.01	0.99
	95% CI		0.54–1.13	0.62–1.34	0.66–1.43	0.66–1.42	0.69–1.49	0.66–1.46	0.71–1.58	0.68–1.54	0.67–1.53	0.66–1.50
	Sylhet vs Dhaka (OR)	-	1.19	1.37	1.50	1.49	1.51	1.49	1.46	1.45	1.44	1.44
	95% CI		0.76–1.88	0.87–2.18	0.94–2.39	0.94–2.37	0.95–2.20	0.93–2.37	0.91–2.33	0.90–2.32	0.90–2.30	0.90–2.31
Age	45–54 vs 35–44 (OR)	-	-	**1.47** **	**1.58** **	**1.59** **	**1.59** **	**1.54** **	**1.54** **	**1.55** **	**1.55** **	**1.52** **
	95% CI			**1.10–1.96**	**1.18–2.11**	**1.19–2.12**	**1.19–2.13**	**1.15–2.06**	**1.15–2.06**	**1.15–2.08**	**1.15–2.09**	**1.13–2.05**
	55–64 vs 35–44 (OR)	-	-	**2.49** ***	**2.57** ***	**2.56** ***	**2.51** ***	**2.22** ***	**2.21** ***	**2.28** ***	**2.27** ***	**2.21** ***
	95% CI			**1.83–3.38**	**1.89–3.51**	**1.88–3.50**	**1.84–3.43**	**1.60–3.06**	**1.60–3.05**	**1.64–3.18**	**1.63–3.17**	**1.58–3.09**
	65+ vs 35–44 (OR)	-	-	**1.75** ***	**2.13** ***	**2.05** ***	**2.04** ***	**1.55** *	**1.56** *	**1.66** *	**1.66** *	**1.57** *
	95% CI			**1.27–2.41**	**1.54–2.96**	**1.47–2.85**	**1.46–2.84**	**1.05–2.30**	**1.06–2.30**	**1.12–2.47**	**1.12–2.47**	**1.05–2.35**
Education	1–5 vs no (OR)	-	-	-	1.30	1.30	1.26	1.25	1.26	1.22	1.23	1.23
	95% CI				0.98–1.73	0.98–1.74	0.94–1.68	0.93–1.67	0.95–1.69	0.91–1.65	0.91–1.66	0.91–1.66
	6–10 vs no (OR)	-	-	-	**1.42** *	**1.42** *	1.28	1.23	1.26	1.16	1.13	1.12
	95% CI				**1.05–1.92**	**1.05–1.92**	0.94–1.75	0.90–1.68	0.92–1.72	0.84–1.60	0.81–1.58	0.80–1.57
	11+ vs no (OR)	-	-	-	**3.24** ***	**3.24** ***	**2.80** ***	**2.73** ***	**2.81** ***	**2.42** ***	**2.32** ***	**2.25** ***
	95% CI				**2.34–4.49**	**2.33–4.48**	**1.99–3.92**	**1.94–3.84**	**1.99–3.96**	**1.69–3.46**	**1.60–3.38**	**1.54–3.28**
Currently married	Yes vs no (OR)	-	-	-	-	**0.59** *	**0.58** *	0.71	0.70	0.72	0.73	0.75
	95% CI					**0.35–0.99**	**0.34–0.98**	0.41–1.22	0.40–1.21	0.40–1.28	0.41–1.30	0.43–1.33
Having TV	Yes vs no (OR)	-	-	-	-		**1.45** **	**1.42** **	**1.45** **	**1.41** **	1.36	1.37
	95% CI						**1.13–1.86**	**1.10–1.82**	**1.13–1.87**	**1.09–1.83**	0.97–1.91	0.98–1.93
Currently working	Yes vs no (OR)	-	-	-	-	-	-	**0.63** **	**0.64** **	**0.64** *	**0.65** *	**0.66** *
	95% CI							**0.45–0.88**	**0.46–0.89**	**0.46–0.91**	**0.46–0.91**	**0.47–0.93**
Having bicycle	Yes vs no (OR)	-	-	-	-	-	-	-	0.79	0.81	0.82	0.82
	95% CI								0.61–1.03	0.62–1.05	0.63–1.08	0.62–1.08
BMI	Underwght. vs normal (OR)	-	-	-	-	-	-	-	-	0.83	0.83	0.85
	95% CI									0.59–1.15	0.60–1.16	0.61–1.19
	Overwht./obese vs normal (OR)	-	-	-	-	-	-	-	-	**1.85** ***	**1.83** ***	**1.77** ***
	95% CI									**1.34–2.55**	**1.32–2.53**	**1.28–2.46**
Wealth index	Poor vs middle (OR)	-	-	-	-	-	-	-	-	-	1.33	1.34
	95% CI										0.91–1.95	0.92–1.95
	Rich vs middle (OR)	-	-	-	-	-	-	-	-	-	1.41	1.38
	95% CI										0.96–2.06	0.94–2.03
Hypertension	Yes vs no (OR)	-	-	-	-	-	-	-	-	-	-	**1.30** *
	95% CI											**1.00–1.69**

***p≤0.001;

**p≤0.01;

*p≤0.05.

**Table 5:** Results of simple and multivariable logistic regression analyses for female diabetes in Bangladesh.

		IW	IIW	IIIW	IVW	VW	VIW	VIIW	VIIIW	IXW
Residence	City corporation vs rural (OR)	**2.72** ***	-	**2.75** ***	**2.07** ***	**1.71** **	**1.84** ***	**1.48** *	1.35	1.30
	95% CI	**1.98–3.74**		**1.99–3.82**	**1.46–2.93**	**1.20–2.45**	**1.27–2.64**	**1.01–2.16**	0.92–1.97	0.88–1.91
	Urban vs rural (OR)	**1.71** ***	-	**1.75** ***	**1.55** ***	**1.32** *	**1.40** *	1.23	1.13	1.11
	95% CI	**1.32–2.20**		**1.35–2.26**	**1.19–2.01**	**1.01–1.73**	**1.06–1.84**	0.92–1.64	0.84–1.52	0.83–1.49
Region	Barisal vs Dhaka (OR)	-	1.11	1.27	1.14	1.26	1.24	1.35	1.39	1.40
	95% CI		0.72–1.71	0.82–1.96	0.73–1.77	0.81–1.97	0.80–1.94	0.86–2.13	0.88–2.20	0.89–2.22
	Chittagong vs Dhaka (OR)	-	1.19	1.27	1.28	1.29	1.30	1.26	1.24	1.30
	95% CI		0.90–1.57	0.96–1.69	0.96–1.71	0.97–1.72	0.98–1.74	0.94–1.69	0.92–1.67	0.96–1.75
	Khulna vs Dhaka (OR)	-	**0.57** **	**0.64** *	**0.63** *	**0.63** *	**0.63** *	**0.61** *	**0.62** *	**0.60** *
	95% CI		**0.39–0.84**	**0.44–0.95**	**0.43–0.93**	**0.43–0.94**	**0.42–0.93**	**0.41–0.91**	**0.41–0.92**	**0.41–90**
	Rajshahi vs Dhaka (OR)	-	0.99	1.16	1.15	1.20	1.18	1.18	1.20	1.20
	95% CI		0.72–1.35	0.84–1.59	0.84–1.60	0.87–1.66	0.85–1.63	0.85–1.65	0.86–1.67	0.86–1.67
	Rangpur vs Dhaka (OR)	-	0.73	0.87	0.91	0.97	0.96	0.95	0.96	0.94
	95% CI		0.50–1.06	0.59–1.27	0.62–1.35	0.66–1.44	0.65–1.41	0.64–1.42	0.64–1.43	0.63–1.41
	Sylhet vs Dhaka (OR)	-	0.94	1.08	1.13	1.13	1.15	1.17	1.13	1.19
	95% CI		0.60–1.48	0.68–1.70	0.71–1.78	0.72–1.80	0.73–1.83	0.72–1.89	0.70–1.84	0.73–1.93
Age	45–54 vs 35–44 (OR)	-	-	1.18	**1.31** *	**1.27**	**1.26**	**1.31** *	1.30	1.19
	95% CI			0.91–1.53	**1.01–1.71**	**0.97–1.65**	**0.97–1.64**	**1.00–1.72**	0.99–1.71	0.91–1.57
	55–64 vs 35–44 (OR)	-	-	**1.44** *	**1.83** ***	**1.83** ***	**1.79** ***	**2.00** ***	**1.96** ***	**1.72** ***
	95% CI			**1.09–1.92**	**1.36–2.47**	**1.35–2.46**	**1.33–2.42**	**1.47–2.71**	**1.44–2.66**	**1.26–2.35**
	65+ vs 35–44 (OR)	-	-	1.29	**1.76** ***	**1.69** ***	**1.66** **	**1.87** ***	**1.79** ***	**1.46** *
	95% CI			0.96–1.74	**1.28–2.41**	**1.23–2.33**	**1.20–2.28**	**1.33–2.61**	**1.27–2.51**	**1.03–2.08**
Education	1–5 vs no (OR)	-	-	-	**1.63** ***	**1.54** ***	**1.49** **	**1.37** *	**1.31** *	**1.33** *
	95% CI				**1.27–2.09**	**1.19–1.98**	**1.15–1.92**	**1.05–1.78**	**1.01–1.71**	**1.02–1.74**
	6–10 vs no (OR)	-	-	-	**2.32** ***	**1.99** ***	**1.90** ***	**1.57** **	**1.45** *	**1.44** *
	95% CI				**1.71–3.15**	**1.46–2.72**	**1.39–2.61**	**1.13–2.19**	**1.04–2.03**	**1.03–2.01**
	11+ vs no (OR)	-	-	-	**2.91** ***	**2.41** ***	**2.24** ***	**1.81** *	**1.67** *	**1.69** *
	95% CI				**1.81–4.67**	**1.49–3.89**	**1.38–3.64**	**1.10–2.98**	**1.02–2.75**	**1.02–2.79**
Having TV	Yes vs no (OR)	-	-	-	-	**1.66** ***	**1.62** ***	**1.50** ***	1.20	1.19
	95% CI					**1.32–2.10**	**1.28–2.04**	**1.18–1.92**	0.88–1.64	0.87–1.63
Land ownership	No vs yes (OR)	-	-	-	-	-	1.24	1.22	1.17	1.18
	95% CI						0.99–1.54	0.98–1.52	0.93–1.46	0.93–1.48
BMI	Underwght. vs normal (OR)	-	-	-	-	-	-	**0.58** **	**0.60** **	**0.63** **
	95% CI							**0.41–0.82**	**0.42–0.85**	**0.44–0.90**
	Overwht./obese vs normal OR)	-	-	-	-	-	-	**1.94** ***	**1.89** ***	**1.71** ***
	95% CI							**1.51–2.50**	**1.47–2.43**	**1.32–2.21**
Wealth index	Poor vs middle (OR)	-	-	-	-	-	-	-	1.07	1.07
	95% CI								0.74–1.53	0.74–1.54
	Rich vs middle (OR)	-	-	-	-	-	-	-	**1.61** **	**1.57** *
	95% CI								**1.13–2.29**	**1.10–2.23**
Hypertension	Yes vs no (OR)	-	-	-	-	-	-	-	-	**1.74** ***
	95% CI									**1.39–2.17**

***p≤0.001;

**p≤0.01;

*p≤0.05.

The multivariable results of diabetes in relation to other potential factors provided mixed results (Tables 4 and 5). Higher age groups (45–54, 55–64 and 65+) indicated higher risks of diabetes for both sexes as compared to the reference group of 35–44 years. Higher educational levels for both sexes also indicated higher risks of diabetes with stronger associations in men. In the final model for men (XIM in table 4) and women (IXF in table 5), the risk of diabetes was 2.25 times (OR_11+years of education/men_ = 2.25, 95% CI = 1.54–3.28) and 1.69 times (OR_11+years of education/women_ = 1.69, 95% CI = 1.02–2.79) more as compared to the no education group. Having television was associated with higher risk of diabetes for both men and women. Status of currently working (yes) for men had consistently lower risk of diabetes in all models. Although participants with overweight/obesity had significantly higher risks of diabetes for both sexes, only underweight women had significantly lower risks of diabetes. Rich as compared to middle class women showed significantly higher risks of diabetes only for women but not for men although the risks were elevated. Finally, status of hypertension also remained significantly associated with diabetes for both sexes.

## Discussion

One of the important findings of our study is that the prevalence of diabetes is substantial among the general adults aged 35 years and older in Bangladesh, which is gradually increasing over time. Around one-tenth of the general population (based on our sample) suffered from diabetes. The overall prevalence of diabetes was insignificantly higher in women than men. The insignificant difference in diabetes prevalence between men and women was also reported by other studies in Bangladesh and other countries [21], [28], [33], [37]. However, one study in India reported significantly higher prevalence of diabetes among men than women [24]. Unfortunately, most of the adults with diabetes in our study were unaware of their disease before testing their blood during the survey. These findings indicate that millions of general adults aged 35 years and older are already suffering from diabetes without proper healthcare attention. These findings provide evidence for an increased burden of diabetes in Bangladesh, which in turn significantly contributes to the global burden of disease [3].

Although regional disparities (based on regions of Bangladesh) in diabetes (in terms of odd ratios) changed from one model to another model (Table 4 and 5), we observed higher burdens in some regions than others. For instance, we found significantly higher (for men) or elevated (i.e. marginally significant for women) risks of diabetes in Chittagong division as compared to Dhaka division. In contrast, people from Khulna division had significantly lower (for women) or lesser (marginally significant for men) risks of diabetes compared with Dhaka division. Higher occurrences of diabetes were also observed in Barisal (for both sexes) and Sylhet (for men) divisions. Possible reasons for regional variations are still unknown, so further epidemiological studies are highly recommended. Our findings further suggest that disparities in diabetes prevalence by place of residence are very much dependent on other variables included in the statistical models. For instance, a simple (i.e., unadjusted) logistic regression model clearly demonstrated significant variations of diabetes by place of residence with higher risks in city corporations and small towns as compared to rural populations. These variations declined gradually in the larger statistical models with more potential exposure factors for diabetes. We observed that ‘having TV’ (model VM in table 4) decreased the risk of diabetes in both city corporations and small towns (see VIM in table 4), which made the significant disparities in diabetes prevalence by place of residence insignificant. Such disparities based on further models (IXM to XIM in table 4) became almost negligible for men. We observed similar situations for women, meaning significant variations of diabetes by place of residence (shown in models IF to VIIF) became insignificant when the variable ‘wealth index’ is included into the model (VIIIF in table 5). Some other studies also indicated insignificant difference of diabetes between rural and urban areas [21]. However, one study in India found significantly higher risks of diabetes in the cities than smaller towns and rural areas even in the multivariable analysis [24].

The highest risks of diabetes were found in the age group of 55–64 years, followed by 65+ years and 45–54 years. Higher levels of education and social class (i.e. wealth) are identified as risk factors for diabetes. These results are found to be consistent with the findings of other studies in Bangladesh and neighbouring countries [12], [21], [28], [30]. An elevated risk of diabetes may be associated with economic development and affluent lifestyles (e.g. intake of excess caloric foods) and less physical activities. Notable changes in lifestyles, diets, exercises, and environmental factors could also be responsible for increasing trend of diabetes and other non-communicable diseases [5], [13]. A remarkable improvement in road communication, electrification and mechanisation of the agriculture industry, automation of daily activities, availability of fast foods, popularisation of television and increased computer usage in rural areas have minimised the physical activity of general people in South Asia including Bangladesh [6], [12], [29]. The positive association of wealth and diabetes could be related with the increasing ability to purchase food, energy intake, and sedentary lifestyles leading to obesity. In contrast, socioeconomically poor people are engaged in physically vigorous activities [12]. Due to the progressive economy and rapid urbanisation in Bangladesh, an increasing number of rural people are migrating to urban areas and changing their professions from physically vigorous agricultural activities to light/sedentary professions. Moreover, globalisation (and super marketization) of processed and fatty foods, particularly in urban areas, may lead to obesity and diabetes in low- and middle-income countries [41].

Physical activity is reported as a protective factor for diabetes [1], [30], [37]. Although our study did not have any direct measurement of physical activity, we used some other mobility indicators like being currently employed, having a bicycle in a household, and having a TV in household. We may assume that household having bicycle and respondent’s status of currently working may increase the chance of mobility. In contrast, households having TV could increase the chance of watching TV and hence would follow sedentary lifestyles. The positive association of watching TV with intake of unhealthy snacks, sugary beverages and fast foods can be partly explained by the increased exposure to food and beverage advertisements [1]. It is interesting to note that households having bicycles was associated with lower risk of diabetes among men in Bangladesh. This may be attributed to the cultural norm of Bangladesh, since riding bicycle is only popular among men but not among women. The lower risk of diabetes in people, who are currently working (for men), may support the negative association between physical mobility and diabetes. Finally, the increased risk of diabetes among the respondents having TV also signifies the positive association of diabetes and sedentary lifestyles.

Like other studies [6], [28], [30], overweight/obesity was found to be associated with significantly higher risk of diabetes. This might be because overweight/obese subjects are generally more insulin resistant as compared to other groups of BMI [37]. The significant association between hypertension and diabetes is also reported by many studies [21], [22], [30], [32], [33], [36], [37]. Since the prevalence of obesity and hypertension are highly prevalent and gradually increasing in low- and middle-income countries including Bangladesh [6], [26], public health strategies should combine both diabetes and hypertension together.

Although we did not perform in-depth analyses for subjects with pre-diabetes, the prevalence is already high in Bangladesh including other neighbouring countries. These findings highlight the potential of rapid progression of diabetes in South Asia [12]. Generally Asians including Bangladeshi people experience early-onset diabetes (at least one decade earlier), suffer longer and die sooner and the overall consequences are more severe for them as compared to white populations in developed countries. They have increased susceptibility to type 2 diabetes because of early pancreatic β-cell failure and prominent central obesity [6]. They also suffer more from high blood pressure and heart disease than other people with the same BMI [6], [14]. Therefore, they are more insulin resistant than Europeans and Caucasians [1], [6], [8], [10], [12]. These situations in Asia may be explained as ‘epigenetic risk’ where environmental triggers are in higher effect.

Since diabetes, hypertension and obesity are substantially modifiable through lifestyle and dietary changes, preventive strategies could easily be developed through public health interventions [1], [14], [18], [26]. Preventive approaches might be more cost effective than curative approaches [6], [10] in Bangladesh because of additional burden (through treatment cost and loss of productivity) for many families with diabetes. Health education that promotes and modifies lifestyles through dietary changes and increased physical activities are also beneficial for both general and risk groups [38], [42]. Training of patients for managing their diabetes by avoiding fast and fatty foods and by increasing their physical activities is as necessary as capacity building of healthcare providers for early detecting and treating of diabetes. Furthermore, health education at schools and food labeling based on fat content is recommended to reduce both the national and the global burden of diabetes [10]. Early diagnosis of diabetes and control of hyperglycaemia and hypertension are also important. Public awareness programmes of diabetes and obesity including their complications as well as capacity building of healthcare providers through training and education might be useful to curve the epidemic of diabetes and obesity [10], [18]. Provision of incentives for companies to produce healthy food containing less fat is also necessary [18]. Since urbanisation with unplanned settlement development was found as a potential risk factor for diabetes in low- and middle-income countries including Bangladesh, we suggest policy support for proper urban planning especially in fast growing megacities of low- and middle-income countries. A health promoting urban infrastructure involving urban green parks to allow residents for healthy physical exercises is considered essential for a better urban population health. We further suggest longitudinal cohort studies to understand the risk factors as well as to understand the prognosis and progression of diabetes. It should be noted that longitudinal cohort studies can provide more reliable evidence than cross-sectional studies. Until now, there is no longitudinal cohort study available in Bangladesh [14].

### Strength and limitations

One of the remarkable strengths of this study is the large sample size, which represents the general adult (aged 35+ years) population in Bangladesh. To our knowledge, this is the first survey, which collected diabetes-related information from the general population. Hence all the findings of this study are very much useful for Bangladesh. The second important strength of the study is the focus on geographical disparities in diabetes prevalence divided into large cities, small towns and rural areas. Such studies are limited although many studies compared rural-urban disparities in diabetes prevalence are already conducted in various countries. Sex-specific analyses of the data also increased the overall strength of the study. However, our study is not free from limitations. Some of the important risk factors for diabetes such as history of diabetes [12], [29], improved measures of physical activities (instead of currently working), sedentary lifestyles (instead of having TV and bicycle) and cholesterol [8] may alter our findings. Our findings also limited our interpretation, as we cannot comment on the cause-effect relationships because of the cross-sectional study type. We merged overweight and obese groups because of limited sample in the obese group, although the risk of diabetes was much higher in the obese group as compared to the overweight group.

Despite these limitations, our empirical findings may lead us to conclude the following: The prevalence of diabetes was found to be almost similar in both sexes. The substantial burden of diabetes along with hypertension and obesity (metabolic syndrome) are both health and economic issues and demands urgent preventive measures. The sex-specific risks factors (i.e. covariates) and their strengths of associations with diabetes may vary sometimes. Particularly disparities in diabetes prevalence by place of residence may differ depending on the potential variables included in the model. The risks of diabetes differ from region to region, with higher risks in Chittagong division and lower risk in Khulna division as compared to Dhaka division. Advancing age, higher level of education and wealth, overweight/obesity and hypertension were independent risk factors for diabetes. Increasing obesity, less physical mobility and large cities (due to rapid urbanization) are the driving factors for the substantial burden of diabetes in Bangladesh.

## Supporting Information

Data S1 **Data used for analysis.**
(7Z) Click here for additional data file.
